# Circulating Lipoproteins in Subjects with Morbid Obesity Undergoing Bariatric Surgery with Gastric Bypass or Sleeve Gastrectomy

**DOI:** 10.3390/nu14122381

**Published:** 2022-06-08

**Authors:** Jan O. Aaseth, Helge Rootwelt, Kjetil Retterstøl, Knut Hestad, Per G. Farup

**Affiliations:** 1 Department of Research, Innlandet Hospital Trust, P.O. Box 104, N-2381 Brumunddal, Norway; knut.hestad@inn.no (K.H.); per.farup@ntnu.no (P.G.F.); 2 Department of Health and Nursing Science, Faculty of Health and Social Sciences, Inland Norway University of Applied Sciences, N-2418 Elverum, Norway; 3 Department of Medical Biochemistry, Oslo University Hospital, N-0424 Oslo, Norway; hrootwel@ous-hf.no; 4 Department of Nutrition, Institute of Basic Medical Sciences, University of Oslo, N-0317 Oslo, Norway; kjetil.retterstol@medisin.uio.no; 5 Lipid Clinic, Oslo University Hospital, N-0424 Oslo, Norway; 6 Department of Clinical and Molecular Medicine, Faculty of Medicine and Health Sciences, Norwegian University of Science and Technology, N-7491 Trondheim, Norway

**Keywords:** dyslipidemia, cholesterol, HDL, LDL, lipoprotein(a), morbid obesity, gastric bypass, gastric sleeve

## Abstract

The efficacy of various bariatric procedures on the mitigation of the obese dyslipidemia remains debated, and the impact of these measures on lipoprotein(a) (Lp(a)) levels is unknown. In this study we aimed to compare the two most commonly used procedures: gastric bypass (RYGB) and sleeve gastrectomy (SG). Adult patients with morbid obesity were assigned to receive either RYGB or SG. The levels of non-HDL cholesterol, LDL/HDL-ratio and Lp(a) at examinations conducted 6 and 12 months postoperatively were determined and compared to preoperative levels to estimate the efficacy of the two surgical methods. All results 6 and 12 months after surgery were used in the comparisons with the preoperative results. A linear mixed regression model for repeated analyses was used. The Lp(a) and the non-HDL cholesterol levels were considerably reduced in the RYGB group, in contrast to the minor changes in the SG group. In addition, the LDL/HDL ratio was significantly more reduced in the RYGB group when compared to the SG group. Conclusively, RYGB was found to be more efficient than SG for the mitigation of obese dyslipidemia, including preoperative high Lp(a)-levels. This might have important individual and societal implications, especially regarding the potential to reduce the risk of cardiovascular disease and the related societal costs.

## 1. Introduction

Obesity has become a worldwide epidemic in recent decades, accompanied by significant negative health effects. Globally, approximately 40% of the adult population were overweight in 2016, and 13% were obese [1].

It is known that obesity is associated with cardiovascular risk factors, such as hypertension, dyslipidemia, diabetes mellitus type 2 (T2DM) and chronic inflammation [2]. An increased risk of morbidity and mortality from cardiovascular disease (CVD) is well-documented in obese patients [3,4]. Bariatric surgery is considered an effective and safe treatment; however, it is reserved for patients with severe obesity, defined as a body mass index (BMI) > 40 kg/m^2^ or BMI > 35 kg/m^2^ in the presence of severe co-morbidities such as hypertension or diabetes 2 (T2DM) [5]. The two most common methods for bariatric surgery today are Roux-en-Y gastric bypass (RYGB) and sleeve gastrectomy (SG). Bariatric surgery results in significant weight loss, reduced all-cause mortality and reduced mortality from cardiovascular disease (CVD) [4,6]. However, the differences in outcome following different surgical techniques are insufficiently explored. The reduction in CVD mortality has, in part, been ascribed to a postoperative improvement of the dyslipidemia accompanying morbid obesity [4,6,7]. These lipid disturbances are characterized by reduced levels of high-density cholesterol (HDL) and increased levels of low-density cholesterol (LDL), in addition to elevated fasting values for triglycerides (TG) [3], a pattern that appears to be ameliorated by bariatric surgery [6]. A lot of research has been carried out concerning whether advanced lipoprotein speciation can improve estimation of CVD. However, in clinical settings, LDL and non-HDL cholesterol levels, the LDL/HDL ratio or the ratio between apolipoprotein B and apolipoprotein A1 (the ApoB/ApoA1-ratio) are routinely used in risk estimations [8]. Additional information can be obtained by adding lipoprotein(a) (Lp(a)) determinations into the risk estimations [9]. The impact of bariatric surgery on the circulating levels of the atherogenic lipoprotein Lp(a) is still incompletely investigated.

The aim of the present study was to trace changes in the lipemic profile of morbidly obese individuals after bariatric surgery, with a focus on the LDL/HDL and non-HDL cholesterol, in addition to assessments of the Lp(a) concentrations. Especially, we intended to explore if observed changes were dependent upon the surgical method used (RYGB or SG).

## 2. Materials and Methods

### 2.1. Study Design and Participants

This retrospective cohort study made use of data and analyses of blood plasma samples collected during the prospective cohort study MO-BiPS (Morbid Obesity—BioPsychoSocial impacts) [10,11].

Subjects with morbid obesity, defined as BMI > 40 kg/m^2^ or BMI > 35 kg/m^2^, with obesity-related complications and referred to the obesity unit at Innlandet Hospital Trust, Gjøvik, Norway for evaluation were included in the research program, provided they were eligible for bariatric surgery [5,10]. Exclusion criteria were severe somatic and psychiatric disorders not related to obesity, alcohol or drug addiction and previous major abdominal surgery.

Preoperatively, the participants went through a behavioral intervention for six months with advice on dietary habits and physical activity. Three weeks before bariatric surgery, they followed a strict “crispbread diet” with a maximum daily intake of 4200 kJ [11]. During the preoperative lifestyle intervention period, the average BMI of the participants was reduced from 42.0 to 38.8 kg/m^2^ [11].

After the lifestyle intervention, bariatric surgery was performed in accordance with current guidelines either as a standard RYGB or as a regular SG procedure [5]. The allocation to the operative method was carried out by the surgeons. Six and twelve months after surgery, there were follow-up visits with blood sampling and examination.

Demographic and anthropometric data, including age (years), sex (male/female), height (m), body weight (kg) and body mass index (BMI; kg/m^2^) were available from the prospective MO-BiPS cohort study. Data from the visits immediately before surgery and from the follow-up visits six and twelve months after surgery were used in the present study.

### 2.2. Blood Sampling and Biochemical Analyses

During the hospital visits, venous blood samples were obtained under fasting conditions from the cubital vein. Blood was sampled in sterile Vacuette^®®^ blood collection tubes using the Vacuette^®®^ blood collection system (Greiner Bio-One, Merck, Darmstadt, Germany). Concentrations of HbA_1c_ were determined immediately after blood sampling. The other blood samples were immediately centrifuged at 2200× *g* for 10 min at 4 °C. Analyses of TG, cholesterol, LDL cholesterol and HDL cholesterol were undertaken at the hospital laboratory in Gjøvik, within approximately one hour after blood sampling by use of Cobas c501 analyzer (Roche 103 Diagnostics, Basel, Switzerland).

Plasma samples for analyses of ApoA1, ApoB and Lp(a) were transferred to micro tubes from Sarstedt^®®^ (Micro tubes, PP-nr 72.664) and kept frozen at −70 °C until analysis.

The concentrations of ApoA1, ApoB and Lp(a) were determined with the accredited methods used at the Department of Medical Biochemistry, Rikshospitalet, Oslo University Hospital on the Cobas 8000 c702 instrument (Hitachi High-Technologies Corporation, Tokyo, Japan) using the HDLC4, LDLC3 and LPA2 kits (Roche Diagnostics GmbH, Mannheim, Germany), respectively.

### 2.3. Statistics

Descriptive data are reported as mean (SD) and number with proportion (%). A t-test was used for the unadjusted comparisons between the groups. All results 6 and 12 months after surgery were used in the comparisons with the results before surgery. A linear mixed regression model for repeated analyses was used for the analyses. Changes were reported as estimated coefficients (B values) with 95% confidence intervals and p-values. The analyses were performed with IBM SPSS Statistics for Windows, version 27.0 (IBM Corp., Armonk, NY, USA).

### 2.4. Ethics

Written informed consent was obtained from all participants before inclusion in the study, which was approved by The Norwegian Regional Committee for Medical and Health Research Ethics, PB 1130, Blindern, 0318 Oslo, Norway (reference number 2012/966) and the study was conducted according to the Declaration of Helsinki.

## 3. Results

The study included 159 subjects. Seven subjects with severe comorbidity were erroneously included and therefore excluded. One hundred and twenty-one subjects completed the lifestyle intervention and underwent bariatric surgery. Data from 111 subjects (women/men: 88/23 with a mean BMI of 38.8 (SD 3.8) kg/m^2^) with at least one measurement of lipids before or after surgery were included in the analyses. Lipid analyses were available from 99–101, 97 and 88–90 subjects before, 6 months and 12 months after surgery, respectively. In the total cohort two patients used statin [11], one in each surgical group. Table 1 shows the participant characteristics before surgery. The characteristics are given for all participants and divided by the type of surgery, with comparisons between the groups.

BMI and CRP were significantly reduced 6–12 months after surgery without significant differences between the types of surgery. Except for total cholesterol and LDL, all lipids were significantly improved after surgery. The differences between the surgery methods in the changes in TG, cholesterol, non-HDL, LDL, LDL/HDL ratio, and Lp(a) are all statistically significant and in favor of RYGB (Table 2). Table 2 shows all changes in BMI, CRP and lipids after surgery with comparisons between the two types of surgery. Notably, the postoperative difference in mean LDL values between RYGB and SG surgery was as large as 0.47 mmol/L.

**Figure 1:**
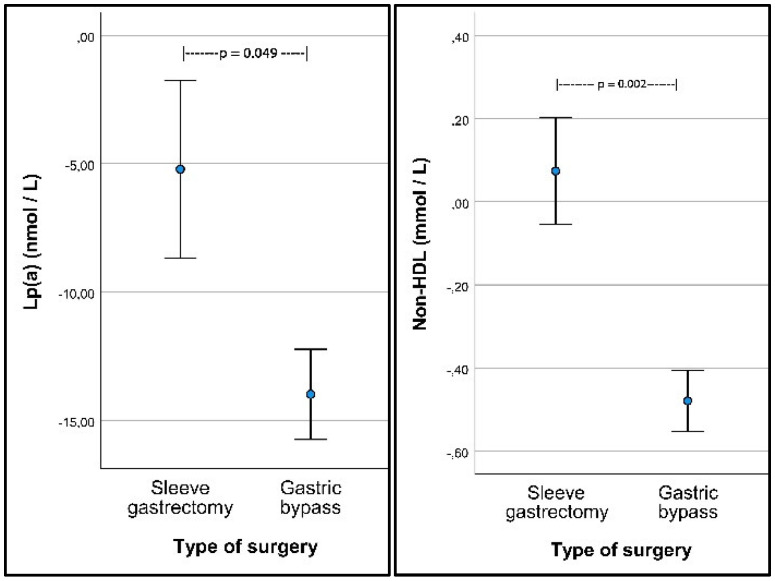
Changes in Lp(a) and non-HDL (mean with SEM) from before to 6–12 months after surgery in subjects operated with sleeve gastrectomy and Roux-en-Y gastric bypass with comparisons between the groups.

The primary focus in our study was the effect of the two types of surgery on changes in Lp(a) and non-HDL. The favorable changes in these variables are statistically significant in favor of RYGP. Figure 1 shows the changes in these variables depending on the type of surgery and with comparisons between the two surgical groups.

Table 3 shows all associations between the independent variables (BMI, diabetes, sex and age) and the lipid species investigated. Of note, BMI was significantly and positively associated with all lipids except for Lp(a), total cholesterol, and ApoA1. Among the independent variables the diagnosis of diabetes was only associated with the triglyceride values. Increasing age was associated with total cholesterol and HDL cholesterol.

## 4. Discussion

In the present study in which morbidly obese subjects underwent bariatric surgery with RYGB or SG, we demonstrate substantial postoperative reductions in Lp(a) and non-HDL cholesterol levels and in the LDL/HDL ratio after the RYGB treatment. The SG procedure resulted in significantly less changes in the same lipid levels.

Whereas the demonstrated effect of RYGB surgery on reducing the circulating levels of Lp(a) (Figure 1) has not been documented before, our observed effects of bariatric surgery on the LDL/HDL ratio are in accordance with previously published studies [12,13,14]. The significant reduction in non-HDL cholesterol after RYGB surgery, which substantially differs from the SG-induced change (Table 2), is of special relevance for patients with previous CVD. Thus, in a recent study of 2638 such patients, a major adverse cardiovascular event occurred in 12% of the patients who had undergone bariatric surgery vs. in as much as 20% of matched controls during a median follow-up period of 4.6 years [15]. In this latter study, more than 80% of the bariatric procedures were performed by RYGB. A better understanding of the importance of the surgical method for reducing cardiovascular risk might lead to further improvements in the surgical approach, for example, by developing techniques that can exert additional benefits by reducing intestinal bile acid reabsorption [16].

As for the atherogenic lipid Lp(a), there are scant data in the literature on the impact of bariatric surgery. Our observed significant reduction in Lp(a) after RYGB, which is apparently *not* related to the weight reduction (Table 3), may allow for new approaches in the research on Lp(a)-lowering mechanisms. It is known that statins or other conventional lipid-lowering agents have minimal or no effects on Lp(a) [17]. In a previous small study on premenopausal women (*n* = 69) treated with bariatric surgery, researchers observed a reduction in Lp(a) following RYGB, in contrast to a negligible change after SG [18]. Our observations together with their results are intriguing and call for further studies to disclose mechanisms of Lp(a) reduction. It might be speculated whether the reduction in Lp(a) induced by the RYGB procedure is related to post-surgical physiological conditions, which include changes in the hepatobiliary metabolism and/or changed fate of components in secreted bile. Here it is to be noted that resins used to reduce serum LDL cholesterol by inhibiting bile acid reabsorption are apparently inefficient in reducing serum levels of Lp(a) [19]. However, recent research has identified new aspects in the biological effects of bile acids, including a possible role as modulators for the farnesoid X receptor (FXR), a receptor which, upon activation, appears to affect lipid metabolism in metabolic disorders [20]. In this context, our observation of a significant reduction in Lp(a) following an RYGB but not SG surgery should be highly relevant for the huge efforts in ongoing research to obtain a more precise understanding of the physiological regulation of Lp(a).

The impact of bariatric surgery on LDL levels might be considered rather modest when compared with the results of preoperative lifestyle intervention [11], in which during a six-month period, a reduction of approximately 15% in both LDL and TG levels [11]. This observation emphasizes the favorable effects of lifestyle intervention, either as a preoperative measure or on its own. In addition, a reasonable interpretation is that the lowering of LDL beyond the RYGB-achieved reduction cannot be expected.

Upon admission to surgery, the subjects in the present study had low levels of HDL (1.1 mmol/L) and ApoA1 (1.1 g/L), which is typical for obese dyslipidemia [21]. The observed lowering of the LDL/HDL ratio, which in part depends on increased postoperative levels of HDL (and apoA1) is in accordance with previous observations [22]. Prognostically, the increments in HDL and ApoA1 might be of importance since one of the central functions of ApoA1 is suggested to be the transportation of cholesterol from macrophages in the arterial wall to the liver for excretion [23].

Relevant for a cardioprotective role of weight loss and bariatric surgery is a reduced general inflammation [24], as is illustrated by the CRP reduction from 7.4 to 4.4 mg/L after the lifestyle intervention [11] and further to approximately 1.4 mg/L postoperatively, irrespective of the surgical method used.

### Strengths and Limitations

The study population in the present single-center study, although unselected, is considered representative of Norwegian patients referred for evaluation with regard to bariatric surgery. The treatment followed national and international guidelines with a lifestyle intervention before surgery using a standard approach [11], and regular follow-ups after surgery. A spectrum of lipoproteins known to be related to obesity was analyzed. However, it can be claimed that the unbalanced male-to-female distribution in favor of females limits external validity. As for statin use, exact doses were not registered for subjects on such treatment.

## 5. Conclusions

In the present study on postoperative results of bariatric surgery, non-HDL cholesterol and Lp(a) levels were considerably reduced in the RYGB treated group. These changes were more significantly pronounced in the RYGB than in the SG group. The mechanisms behind the reduction in Lp(a) after RYGB are unknown and call for further research. Conclusively, a gastric bypass was found to be superior to sleeve gastrectomy for mitigating obese dyslipidemia. This might have important individual and societal implications, especially in reducing the risk of cardiovascular disease and related societal costs.

## Figures and Tables

**Table 1:** Characteristics of all participants before bariatric surgery and divided according to the type of surgery with Roux-en-Y gastric bypass or sleeve gastrectomy.

Characteristics (Number (n) If Less Than 111)	Result Mean (SD) or n (%)	Results Gastric Bypass (n 90)	Results Sleeve Gastrectomy (n 21)	Statistics * p-Values
Sex (male/female)	23 (21%)/88 (79%)	22 (24%)/68 (76%)	1 (5%)/20 (95%)	0.069
Age (years)	43.0 (8.2)	42.9 (8.6)	43.3 (6.8)	0.825
BMI (kg/m^2^)	38.8 (3.8)	39.1 (3.6)	37.2 (4.1)	0.825
Diabetes	19 (17%)	17 (20%)	2 (9.5%)	0.351
HbA1c (%) (n 101)	5.48 (0.87)	5.46 (0.67)	5.55 (1.43)	0.689
CRP (mg/L)	4.41 (3.92)	4.36 (4.09)	4.62 (3.14)	0.783
Triglycerides (mmol/L) (n 101)	1.33 (0.52)	1.38 (0.54)	1.09 (0.35)	**0.005**
Cholesterol (mmol/L) (n 101)	4.36 (0.88)	4.36 (0.91)	4.38 (0.77)	0.930
HDL (mmol/L) (n 101)	1.11 (0.30)	1.06 (0.29)	1.30 (0.26)	**0.001**
Non-HDL (mmol/L) (n 101)	3.26 (0.89)	3.08 (0.77)	3.30 (0.92)	0.327
LDL (mmol/L) (n 101)	2.78 (0.82)	2.81 (0.84)	2.69 (0.71)	0.548
LDL/HDL ratio (n 101)	2.73 (1.16)	2.86 (1.19)	2.17 (0.84)	**0.016**
ApoA1 (g/L) (n 99)	1.14 (0.19)	1.12 (0.19)	1.22 (0.17)	**0.032**
ApoB (g/L) (n 99)	0.86 (0.21)	0.86 (0.21)	0.85 (0.21)	0.778
ApoB/ApoA1 ratio (n 99)	0.77 (0.23)	0.79 (0.24)	0.70 (0.19)	0.126
Lp(a) (nmol/L) (n 99)	53.6 (63.0)	52.4 (61.4)	58.7 (70.8)	0.700

* Statistics for differences between the groups treated with gastric bypass and sleeve gastrectomy.

**Table 2:** Changes in BMI, CRP and the lipid profile from before to 6–12 months after bariatric surgery. The analyses were performed with a linear mixed regression model adjusted for age and gender.

Dependent Variables	Changes * † All Subjects	Changes * Gastric Bypass	Changes * Sleeve Gastrectomy	Change * Differences (Bypass Minus Sleeve)
BMI (kg/m^2^)	−9.57 (−10.08; −9.07) ***p* < 0.001**	−9.70 (−10.26; −9.13) ***p* < 0.001**	−9.04 (−10.21; −7.87) ***p* < 0.001**	−0.65 (−1.95; 0.64) *p* = 0.320
CRP (mg/L)	−2.99 (−3.52; −2.45) ***p* < 0.001**	−3.02 (−3.60; −2.43) ***p* < 0.001**	−2.85 (−4.11; 1.26) ***p* < 0.001**	−0.16 (−1.55; 1–23) *p* = 0.819
Triglycerides (mmol/L	−0.35 (−0.42; −0.28) ***p* < 0.001**	−0.38 (−0.46; −0.31) ***p* < 0.001**	−0.18 (−0.34; −0.02) ***p* = 0.026**	−0.20 (−0.38; −0.03) ***p* = 0.025**
Cholesterol (mmol/L)	−0.02 (−0.16; 0.12) *p* = 0.752	−0.12 (−0.28; 0.03) *p* = 0.103	0.43 (0.11; 0.74) ***p* = 0.008**	−0.55 (−0.90; −0.20) ***p* = 0.002**
HDL (mmol/L)	0.36 (0.32; 0.40) ***p* < 0.001**	0.36 (0.31; 0.40) ***p* < 0.001**	0.39 (0.29; 0.49) ***p* < 0.001**	−0.03 (−0.14; 0.08) *p* = 0.548
Non-HDL (mmol/L)	−0.38 (−0.52; −0.25) ***p* < 0.001**	−0.48 (−0.62; −0.33) ***p* < 0.001**	0.04 (−0.26; 0.34) *p* = 0.789	−0.52 (−0.86; −0.19) ***p* = 0.002**
LDL (mmol/L)	−0.12 (−0.25; 0.004) *p* = 0.057	−0.21 (−0.35; −0.07) ***p* = 0.003**	0.26 (−0.03; 0.54) *p* = 0.076	−0.47 (−0.78; −0.15) ***p* = 0.004**
LDL/HDL ratio	−0.80 (−0.94; −0.66) ***p* < 0.001**	−0.90 (−1.05; −0.75) ***p* < 0.001**	−0.35 (−0.67; −0.04) ***p* = 0.027**	−0.54 (−0.89; −0.20) ***p* = 0.002**
ApoA1 (g/L)	0.23 (0.20–0.26) ***p* < 0.001**	0.23 (0.19; 0.26) ***p* < 0.001**	0.26 (0.18; 0.33) ***p* < 0.001**	−0.03 (−0.11; 0.05) *p* = 0.463
ApoB (g/L)	−0.04 (−0.07; −0.01) ***p* = 0.007**	−0.06 (−0.09; −0.02) ***p* = 0.001**	(−0.06; 0.09) *p* = 0.706	−0.07 (−0.15; 0.01) *p* = 0.086
ApoB/ApoA1 ratio	−0.16 (−0.19; −0.13) ***p* < 0.001**	−0.17 (−0.21; −0.14) ***p* < 0.001**	−0.11 (−0.18; −0.05) ***p* = 0.001**	−0.06 (−0.14; 0.02) *p* = 0.134
Lp(a) (nmol/L)	−12.3 (−15.6; −9.0) ***p* < 0.001**	−13.8. (−17.4; −10.2) ***p* < 0.001**	−5.3 (−13.0; 2.4) *p* = 0.174	−8.5 (−17.0; −0.04) ***p* = 0.049**

* Changes are reported as estimated coefficients (B-values) with 95% confidence intervals and *p*-values. † Calculated without interaction between time and type of surgery.

**Table 3:** Predictors of the lipids. Mixed model linear regression with one of the lipids at a time as dependent variables and gender, age, BMI, diabetes, type of surgery and point of time, all simultaneously, as independent variables.

Dependent Variables	Independent Variables *			
	BMI	Diabetes	Sex (Male)	Age
Triglycerides (mmol/L)	0.02 (0.01; 0.04) ***p* = 0.005**	0.31 (0.13; 0.50) ***p* = 0.001**	−0.02 (−0.21; 0.17) *p* = 0.853	−0.003 (−0.01; 0.01) *p* = 0.441
Cholesterol (mmol/L)	0.03 (−0.003; 0.06) *p* = 0.08	0.05 (−0.28; 0.37) *p* = 0.781	−0.33 (−0.66; −0.01) ***p* = 0.046**	0.02 (0.001; 0.03) ***p* = 0.037**
HDL (mmol/L)	−0.014 (−0.025; −0.004) ***p* = 0.006**	−0.02 (−0.16; 0.13) *p* = 0.827	−0.13 (−0.27; 0.02) *p* = 0.087	0.014 (0.007; 0.021) ***p* < 0.001**
Non-HDL (mmol/L)	0.04 (0.01; 0.07) ***p* = 0.006**	0.06 (−0.28; 0.40) *p* = 0.714	−0.21 (−0.55; 0.13) *p* = 0.226	0.002 (−0.01;0.02) *p* = 0.778
LDL (mmol/L)	0.03 (0.005; 0.06) ***p* = 0.022**	−0.03 (−0.34; 0.28) *p* = 0.243	−0.22 (−0.53; 0.09) *p* = 0.164	0.005 (−0.001; 0.02) *p* = 0.518
LDL/HDL ratio	0.06 (0.03; 0.10) ***p* < 0.001**	0.10 (−0.31; 0.51) *p* = 0.637	0.06 (−0.35; 0.47) *p* = 0.764)	−0.02 (−0.04; −0.01) ***p* = 0.034**
ApoA1 (g/L)	−0.004 (−0.012; 0.003) *p* = 0.269	0.04 (−0.06; 0.14) *p* = 0.418	−0.10 (−0.20; 0.002) *p* = 0.055	0.008 (0.003; 0.012) ***p* = 0.001**
ApoB (g/L)	0.009 (0.002; 0.017) ***p* = 0.014**	0.02 (−0.07; 0.11) *p* = 0.646	−0.06 (−0.15; 0.02) *p* = 0.156	0.001 (−0.003; 0.005) *p* = 0.551
ApoB/ApoA1 ratio	0.012 (0.005; 0.019) ***p* = 0.001**	−0.001 (−0.11; 0.09) *p* = 0.673	0.003 (−0.089; 0.094)*p* = 0.953	−0.003 (−0.008; 0.001) *p* = 0.148
Lp(a) (nmol/L)	0.37 (−0.73; 1.47) *p* = 0.505	−0.79 (−34.5; 32.9) *p* = 0.963	13.0 (−20.2; 46.3) *p* = 0.438	0.71 (−0.86; 2.27) *p* = 0.373

* The results are reported as estimated coefficients (B-values) with 95% confidence intervals and *p*-values.

## Data Availability

The raw datasets generated and analyzed during the current study are not publicly available in order to protect participant confidentiality. Case report forms (CRFs) on paper are safely stored. The data were transferred deidentified to SPSS and R-studio for statistical analyses. The data files are stored by Innlandet Hospital Trust, Brumunddal, Norway, on a server dedicated to research. The security follows the rules given by the Norwegian Data Protection Authority, P.O. Box 8177 Dep. NO-0034 Oslo, Norway. The data are available on request to the authors.
